# Phase II study of temozolomide and veliparib combination therapy for sorafenib-refractory advanced hepatocellular carcinoma

**DOI:** 10.1007/s00280-015-2852-2

**Published:** 2015-10-08

**Authors:** Andrew Gabrielson, Anteneh A. Tesfaye, John L. Marshall, Michael J. Pishvaian, Brandon Smaglo, Reena Jha, Karen Dorsch-Vogel, Hongkun Wang, Aiwu Ruth He

**Affiliations:** Division of Hematology and Oncology, Lombardi Comprehensive Cancer Center, Georgetown University Hospital, 3800 Reservoir Road, NW, Washington, DC 20007 USA; Department of Biostatistics and Bioinformatics, Georgetown University Hospital, 3800 Reservoir Road, NW, Washington, DC 20007 USA; Department of Radiology, Georgetown University Hospital, 3800 Reservoir Road, NW, Washington, DC USA

**Keywords:** Refractory, Hepatocellular carcinoma, Temozolomide, Veliparib

## Abstract

**Purpose:**

To determine the antitumor efficacy and tolerability of combination temozolomide (TMZ) and veliparib (ABT-888) in patients with advanced, sorafenib-refractory hepatocellular carcinoma (HCC).

**Methods:**

This single-arm phase II trial enrolled patients with pathologically confirmed, sorafenib-refractory HCC. All patients received 40 mg ABT-888 PO daily on days 1–7 and 150 mg/m^2^ TMZ PO daily on days 1–5 of a 28-day cycle. The primary endpoint was objective response rate (ORR) at 2 months. Secondary endpoints included overall survival (OS), progression-free survival (PFS), and toxicity profile. Tumor response was assessed every 2 cycles using RECIST criteria, and toxicities were assessed using CTCAE v4.03.

**Results:**

We enrolled 16 patients in the first phase of the trial, but the study was discontinued due to a poor ORR; only four patients (25 %) had SD after 2 cycles. Twelve patients (75 %) were taken off study after 2 months of treatment; 10 of these had disease progression. Two patients (13 %) were taken off study due to severe toxicity, and one patient (6 %) died from non-treatment-related liver failure. One patient had SD for 16 months, receiving 11 cycles of therapy before being taken off study. The most common grade 3 treatment-related toxicities included vomiting (*n* = 2), thrombocytopenia (*n* = 2), nausea (*n* = 1), and anemia (*n* = 1). The median PFS was 1.9 months, and median OS was 13.1 months.

**Conclusion:**

The combination of TMZ and ABT-888 is well tolerated in patients with advanced HCC. However, the regimen failed to show survival benefit.

**ClinicalTrials.gov Identifier:**

NCT01205828.

**Electronic supplementary material:**

The online version of this article (doi:10.1007/s00280-015-2852-2) contains supplementary material, which is available to authorized users.

## Introduction

HCC is known as one of the most lethal human cancer types, mostly due to its resistance to chemotherapy and radiation. It is the second most common cause of cancer death in men and the sixth most common in women worldwide; this is largely due to the fact that only 30–40 % of the patients are amenable to curative treatment at diagnosis [1]. Curative treatments include liver resection, transplant, or local ablation. Five-year survival for well-selected patients who undergo curative therapy is 60–70 % [2]. However, most patients are not eligible for curative treatment and have a dismal prognosis. Sorafenib (Nexavar, Bayer/Onyx), a multi-targeted orally active small molecule tyrosine kinase inhibitor, is the first systemic therapy that has shown survival benefit in patients with HCC, prolonging PFS and OS by 2–3 months. However, the sorafenib response rate is less than 5 % [3, 4]. Due to the aggressive nature of HCC, patients with unresectable disease have a median survival of approximately 6–20 months following diagnosis [5]. Although locoregional therapies such as trans-arterial chemoembolization (TACE) and radiofrequency ablation (RFA) may improve outcomes [6, 7], HCC shows resistance to conventional chemotherapy. Thus, there is an urgent need for improved treatment options. One mechanism of tumor resistance to cytotoxic therapy is via repair of damaged DNA, and we attempted to overcome this form of tumor resistance by targeting tumor DNA repair pathways, namely those involving poly(ADP-ribose)-polymerase (PARP) 1 and 2.

DNA-damaging treatments such as cytotoxic chemotherapy are still a treatment mainstay for many patients with cancer. PARP-1 and PARP-2 are nuclear enzymes that become activated in response to DNA damage and facilitate DNA repair [8, 9]. Thus, PARP inhibition will result in less efficient DNA repair following a cytotoxic insult. As cancer cells are genetically unstable, often exhibiting complex karyotypes that include large deletions, insertions, and unbalanced translocations of chromosomal material, these cells are more susceptible than normal tissues to DNA damage-induced cytotoxicity [10, 11]. Deficiencies in mismatch repair and homologous recombination processes are associated with the largest number of malignancies. These deficiencies render cells more dependent on PARP for DNA repair and hence more sensitive to PARP inhibition [12]. PARP-enabled DNA repair may also compensate for the loss of other repair pathways. Thus, significantly increased poly-ADP-ribosylation and PARP expression was found in HCC samples compared with adjacent normal liver tissue from the same patient following surgical excision and analysis [13]. Higher expression of PARP in cancer cells compared with normal cells has been linked to drug resistance and the overall ability of cancer cells to sustain genotoxic stress [14–17].

ABT-888 is an orally bioavailable PARP-1/2 inhibitor that possesses an excellent efficacy and pharmacokinetic profile. ABT-888 has been shown to enhance the antitumor activity of DNA-damaging agents such as temozolomide, irinotecan, cyclophosphamide, and cisplatin [18], and there is great potential for the broad use of ABT-888 in combination with a host of chemo- and radiotherapeutic regimens, making it an attractive agent for clinical development.

TMZ is a well-studied DNA-methylating agent that crosses the blood–brain barrier and is licensed for the treatment of gliomas and frequently used off-label for malignant melanoma and HCC [19]. TMZ is an oral agent with a broad spectrum of antitumor activity and relatively low levels of toxicity. In this study, we present the first phase II trial utilizing the combination of TMZ and ABT-888 in the treatment of advanced, non-transplantable, sorafenib-refractory HCC.

## Materials and methods

### Patients and clinical study design

This was an open-label single-arm phase II trial using TMZ and ABT-888 as second-line treatment in patients with advanced sorafenib-refractory HCC (IRB# 2009-268). Patients enrolled in this study received ABT-888 PO 40 mg daily on days 1–7 and temozolomide PO 150 mg/m^2^ daily on days 1–5 in 28-day cycles. The trial followed a modification of Simon’s two-stage optimal design as implemented by Hanfelt et al. [20]. For the first stage, 16 patients were accrued. If only 0 or 1 of the 16 patients demonstrated disease control during treatment, the treatment would be rejected and the trial stopped. However, if at least 2 patients (13 %) responded to treatment in the first stage, then an additional 33 patients would be recruited to enter into the second stage, for a total of 49 patients in this phase II study.

Eligible patients had to be at least 18 years old, have histologically and cytologically confirmed, radiographically measurable hepatocellular carcinoma, and have demonstrated disease progression on first-line sorafenib therapy. Progression on sorafenib was defined as radiological or clinical progression or documented unacceptable toxicity. Patients also had to be Child-Pugh class A or B and have an Eastern Cooperative Oncology Group (ECOG) performance status score of 0–2 [21]. Other inclusion criteria included adequate bone marrow (absolute neutrophil count ≥1500/mm^3^; and platelets ≥75,000/mm^3^), liver function (aspartate transaminase and alanine transaminase ≤3.0 times the upper limit of normal range unless liver metastases are present, in which case ≤5.0 times the upper limit of normal range), and renal function (creatinine clearance ≥30 ml/min as calculated by the Cockroft-Gault Equation). Exclusion criteria included prior TMZ or PARP inhibitor (ABT-888 or other) treatment, anticipation of major surgery during study, or concurrent malignancy other than HCC.

Within 30 days of enrollment, each patient received an initial clinical evaluation; had laboratory tests performed; and underwent a CT scan of the chest, abdomen, and pelvis, as well as an MRI of the liver. During treatment, patient status was monitored by a complete blood count every 2 weeks for the first two cycles and every 4 weeks thereafter; a complete metabolic panel with serum alpha-fetoprotein (AFP) measured every 4 weeks; clinical evaluation every 2 weeks for first two cycles and every 4 weeks; and radiological imaging every two cycles unless otherwise indicated. Tumor response and progression were determined according to the Response Evaluation Criteria in Solid Tumors (RECIST) [22]. Toxicity was assessed at every visit using Common Terminology Criteria for Adverse Events (CTCAE) version 4.03. Patients were discontinued from the study in the event of disease progression, uncontrolled side effects, loss to follow-up, or withdrawal of consent. The MedStar/Georgetown Institutional Review Board approved the ethical, legal, and social implications of the study.

### Statistical analysis

The primary objective of this single-arm phase II study was to assess the efficacy of ABT-888 and TMZ as a neoadjuvant therapy in HCC in terms of ORR after 2 months of treatment; this was defined as the combined percentage of patients who had CR, PR, and SD. Secondary endpoints included OS (defined as the number of months between a patient’s enrollment and his/her date of death), PFS (defined as the number of months between a patient’s enrollment and his/her radiographically confirmed disease progression or death), the toxicity profile, and correlations between biological markers and tumor response to the combination therapy. Kaplan–Meier methodology was used to analyze PFS and OS, which were reported with a 95 % confidence interval (CI). The log-rank test was used to assess the association between clinical factors and survival. A *P* value <0.05 was considered statistically significant. SAS software version 9.3 (SAS Inc. Cary, N.C.) was used for statistical analysis.

## Results

### Patient characteristics

Between October 2010 and September 2013, 16 patients were accrued to this study. The stable disease status that was needed to continue enrollment past the first phase was achieved in 4 of the 16 patients (25 %), who experienced stable disease after two cycles of treatment. However, due to the poor ORR (CR + PR + SD) observed, accrual to the study was stopped. The demographic and baseline characteristics of the enrolled cohort are shown in Table 1. The median age was 62 (range, 40–76), and the patient group was predominately male (88 %). At baseline visit, all patients had an ECOG performance status of 0 or 1. Eleven patients (69 %) had vascular invasion of their tumor, 10 patients (63 %) had liver cirrhosis, and seven patients (44 %) had tumor thrombosis. Common etiologies of HCC included eight patients (50 %) with viral hepatitis C and five patients (32 %) with viral hepatitis B infection. Patients were also stratified by most recent serum AFP: Seven patients (44 %) had high serum AFP (≥500) and nine patients (56 %) had low AFP (<500).

**Table 1:** Demographics and baseline characteristics of the HCC patient cohort

Characteristic	Variables	*n* (%)
Age	Media (mix–max)	62 (40–76)
Gender	Female	2 (12 %)
	Male	14 (88 %)
Ethnicity	African American	7 (44 %)
	Caucasian	5 (32 %)
	Asian	2 (12 %)
	Other	2 (12 %)
Risk factor	Hepatitis C	8 (50 %)
	Hepatitis B	5 (32 %)
	Non-alcoholic steatohepatitis	2 (13 %)
	Alcoholism	1 (6 %)
Cirrhosis	Yes	10 (63 %)
	No	6 (37 %)
Child-pugh class	A	12 (75 %)
	B	4 (25 %)
Vascular invasion	Yes	11 (69 %)
	No	5 (31 %)
Serum alpha-fetoprotein	High (>500)	7 (44 %)
	Low (<500)	9 (56 %)
Cellular differentiation	Well	5 (31 %)
	Moderate	8 (50 %)
	Poor	3 (19 %)
Tumor thrombosis	Yes	7 (44 %)
	No	9 (56 %)
ECOG	0	2 (12 %)
	1	14 (88 %)

### Toxicity

Most patients participating in the trial tolerated the combination therapy well, with most toxicities confined to grade 1 or 2 events (Table 2). The most common grade 1 or 2 adverse events were fatigue (50 %), thrombocytopenia (25 %), and nausea (19 %). Five patients (31 %) developed grade 3 or 4 adverse events: two counts of thrombocytopenia, two counts of severe vomiting, and one count each of liver failure, peritoneal bleed, fatigue, nausea, and neutropenia (Table 2). None of the enrolled patients died of treatment-related toxicities.

**Table 2:** Adverse events classified by CTCAE grade in patients receiving combination treatment

Adverse clinical event	Number of Patients (%)		
	Grade 1	Grade 2	Grade 3
Fatigue	2 (13)	6 (38)	1 (6)
Nausea	3 (19)	0 (0)	1 (6)
Vomiting	2 (13)	0 (0)	2 (13)
Neutropenia	0 (0)	1 (6)	1 (6)
Thrombocytopenia	1 (6)	3 (19)	2 (13)
Peritoneal bleeding from tumor rupture	0 (0)	0 (0)	1 (6)
Liver/kidney failure from hepatorenal syndrome	0 (0)	0 (0)	1 (6)

Reason for treatment discontinuation	Number of patients (%)
Disease progression	13 (81)
Grade 3+ clinical toxicity	2 (13)
Death	1 (6)

### Tumor response

Tumor response to treatment was assessed using CT imaging every 2 cycles of treatment (8 weeks). The median number of treatment cycles given to patients enrolled in the trial was two. Ten patients (63 %) had disease progression after 2 or fewer cycles of treatment and were taken off study after their first follow-up imaging scan. Four patients (25 %) had stable disease after 2 cycles of treatment: One patient demonstrated stable disease for 11 cycles of treatment (16 months); one patient had stable disease for 6 cycles (6 months) before being taken off study due to disease progression; one patient had stable disease for 3 cycles (3 months) before being taken off study due to non-treatment-related peritoneal bleeding and tumor rupture; and the last patient with stable disease was taken off study after 4 cycles (4 months) due to disease progression. None of the patients in the trial demonstrated a partial or complete response based on RECIST criteria. Two patients (13 %) were taken off study for severe clinical toxicities prior to receiving their first imaging scan—one patient was taken off study after 4 days of treatment due to severe nausea, vomiting, and a Mallory–Weiss tear, and the other patient died after 1 month of treatment due to progressive liver cirrhosis and hepatorenal syndrome.

### Survival and log-rank analysis

**Fig. 1:**
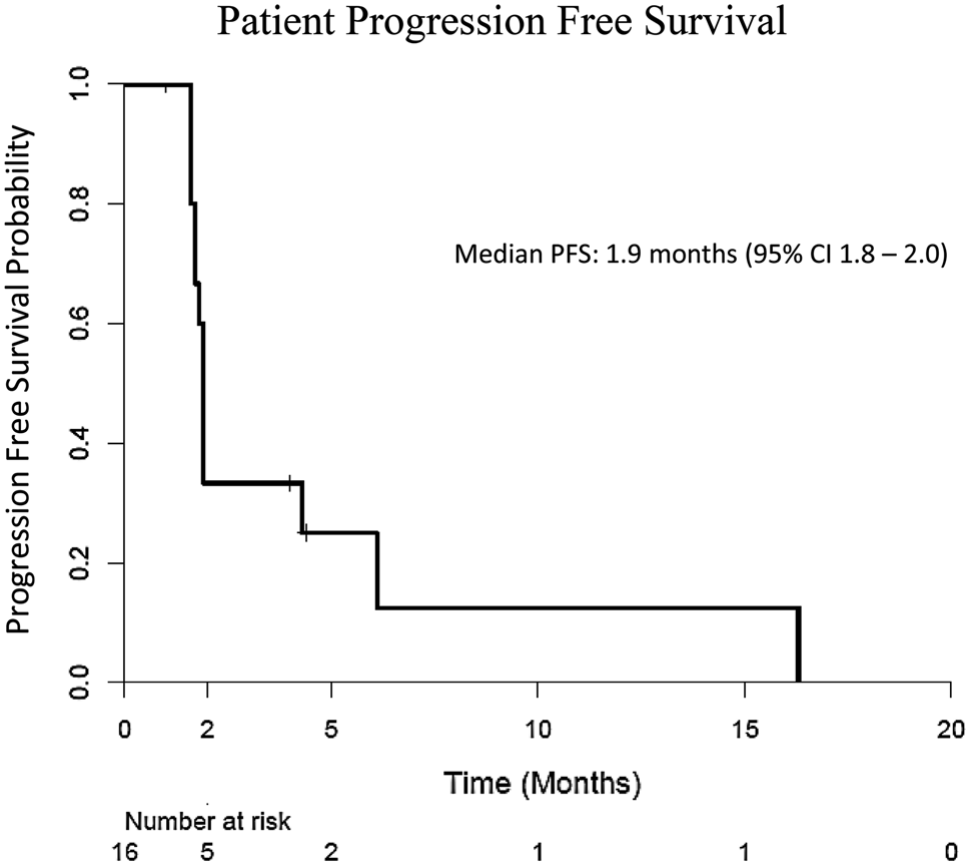
Kaplan–Meier curve for progression-free survival

**Fig. 2:**
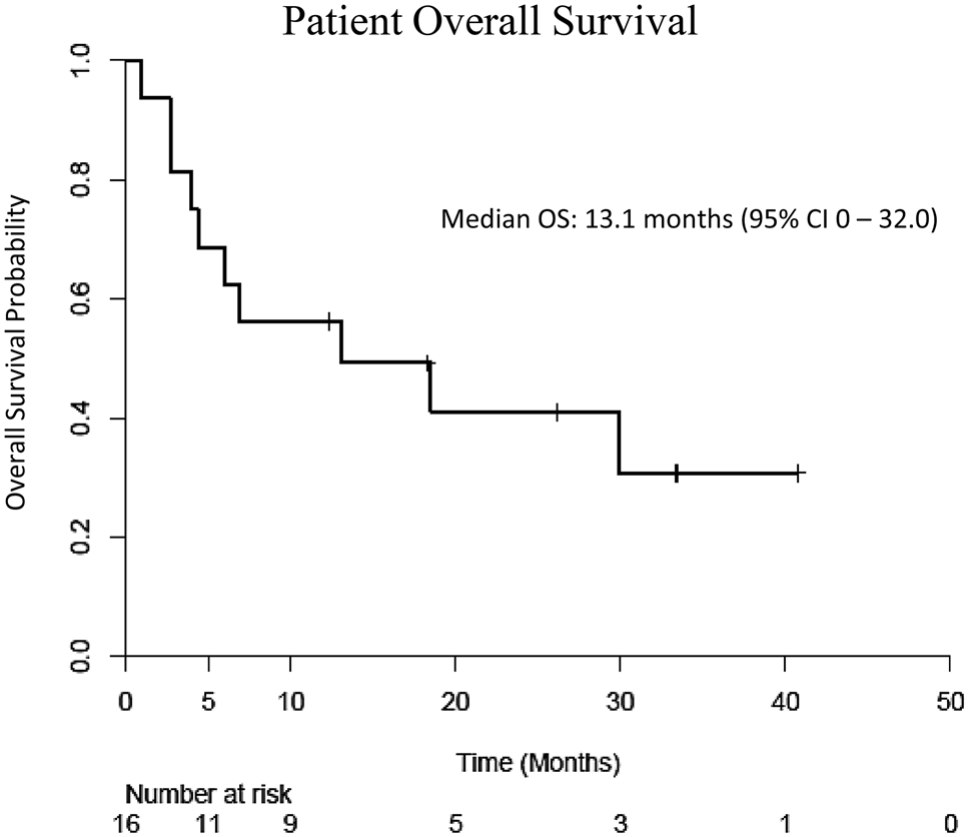
Kaplan–Meier curve for overall survival

Median PFS (Fig. 1) and OS (Fig. 2) for the entire cohort were 1.9 months (95 % CI 1.8–2.0) and 13.1 months (95 % CI 0–32.0), respectively. Patients were stratified by serum AFP level, presence of tumor vascular invasion, Child-Pugh grade, and cellular differentiation to measure their association with OS and PFS. Using the log-rank test, we found that serum AFP, tumor vascular invasion, Child-Pugh grade, and cellular differentiation were not significantly associated with either OS or PFS after receiving the combination therapy (Supplementary Figures 1–8).

## Discussion

FDA approval of sorafenib, the first and only targeted agent for patients with advanced HCC, was an important step forward in the treatment of advanced stage hepatic malignancies. However, sorafenib’s clinical benefit is modest, prolonging both PFS and OS by only 2–3 months with a response rate of less than 5 % [2, 3]. In addition, most patients who receive sorafenib eventually develop progression of their HCC. Given the absence of effective second-line therapies for patients who have progressed on sorafenib, there is an urgent need to find novel treatment combinations.

This study is the first reported in the literature that evaluates the clinical efficacy of the PARP-1/2 inhibitor, ABT-888, and DNA-methylating agent, TMZ, in advanced HCC patients who had disease progression on prior sorafenib treatment. The results of the current study show that ABT-888 and TMZ were well tolerated in patients, although the combination therapy did not demonstrate a survival benefit. The median PFS (1.9 months) and OS (13.1 months) observed in this study are comparable to the PFS and OS observed in patients receiving best supportive care following progression on first-line sorafenib [23]. One patient in our study had marked disease control, with no progression of his HCC for 16 months during treatment. We reviewed the disease course of this patient and found that this patient had stable disease for 11 months on sorafenib prior to enrollment in the current study. Additionally, this patient later enrolled in a phase I trial of a drug with a completely different mechanism of action and continued to demonstrate stable disease for 8 months after discontinuing ABT-888 and TMZ. The prolonged disease control in this patient may reflect the slow progression of his cancer, and may not be attributed to a specific treatment regimen. Tissue from HCC biopsies was obtained from two of the four patients who exhibited disease control and five of the ten patients who demonstrated disease progression after 2 cycles of treatment. However, due to the small number of patients (*n* = 4) who demonstrated disease control, we chose not to proceed with molecular analysis of this tissue but instead to bank it for future better informed analyses.

All patients eventually developed resistance or intolerable clinical toxicities, particularly if the patient had underlying liver cirrhosis. Four patients (25 %) had stable disease after 2 cycles of treatment, but three of these patients would be taken off study due to disease progression or non-treatment-related clinical toxicities after 4 cycles of treatment. Ten patients (63 %) progressed after 2 cycles of treatment, one patient (6 %) died from non-treatment-related liver cirrhosis, and one patient (6 %) was taken off study after 1 week of treatment due to severe refractory nausea and vomiting.

There are several limitations to the current study that must be addressed. Due to the small number of patients recruited, our findings regarding survival and the toxicity profile should be regarded as exploratory rather than confirmatory.

Since 2007, the antitumor efficacy of ABT-888 has been evaluated in 83 clinical trials in a myriad of cancer types, including breast, ovarian, colon, rectal, pancreas, and lung [24]. These studies have improved our understanding of the mechanisms by which cancer cells respond and develop resistance to PARP-1 and PARP-2 inhibitors. Several factors may have contributed to HCC resistance to PARP inhibition therapy in the current study, such as increased expression of DNA repair enzymes. Previous research has indicated enhanced homologous recombination capacity and defective base excision repair as important contributors to ABT-888 and TMZ chemoresistance [25].

Inhibition of PARP-1 activity by ABT-888 limits the cell’s ability to repair single-strand DNA breaks via the base excision repair pathway, which leads to accumulation of double-strand breaks during DNA replication. However, malignant cells with intact BRCA1 and BRCA2 utilize the homologous recombination pathway as an error-free rescue mechanism to repair double-strand breaks and retain genomic stability [26]. BRCA-deficient cells are unable to take advantage of this pathway, and must rely on non-homologous end-joining to repair double-strand breaks. This pathway is prone to erroneous DNA repair and genomic instability, which leads to cancer cell death [26]. Patients receiving the combination therapy who have BRCA-intact tumors would be less likely to exhibit tumor cytotoxicity due to maintenance of homologous recombination pathway activity. As a result, PARP-1/2 inhibition by ABT-888 would be more effective at sensitizing cells to single-strand DNA breaks if patients have BRCA- or homologous recombination-deficient cancer.

Unfortunately, the prevalence of BRCA mutations in HCC is quite low. From whole-genome sequences of over 1000 HCC cases entered into the Catalogue of Somatic Mutations in Cancer (COSMIC) database, rates of BRCA1 and BRCA2 mutations were 0.07 and 1.7 %, respectively [27]. Thus, it remains a challenge to identify patients with BRCA-mutated HCC and test the antitumor efficacy of PARP inhibitors such as ABT-888 in these patients. As mutations in BRCA1 and BRCA2 may predict response to PARP inhibition regardless of cancer type, clinical trials testing the efficacy of PARP inhibitors in cancer with BRCA1 or BRCA2 mutations may need to be extended to all disease types, including HCC. BRCA1 and BRCA2 mutation testing is not a part of the treatment paradigm for HCC, but the increased use of molecular profiling for solid tumors may mitigate the difficulty in identifying candidate patients for PARP inhibition therapy.

Patients with advanced HCC may harbor defects in other genes involved with the repair of double-stranded DNA breaks, such as those involved in the MRE11-RAD50-NBN1 (MRN) complex [28]. However, no studies have demonstrated an association between defects in these genes and sensitivity to PARP inhibition.

Due to evident patient selection difficulties, identifying additional markers that predict which patients will benefit from ABT-888 and TMZ therapy is worthwhile. One candidate marker is the expression level of O^6^-methylguanine DNA methyltransferase (MGMT), a DNA repair enzyme that plays a crucial role in maintaining genomic stability in HCC [29]. This protein is mainly regulated at the epigenetic level through CpG island promoter methylation, which in turn causes functional silencing of the gene [30]. Studies have shown that 50–60 % of HCC tumors are MGMT deficient at the genetic and proteomic level [31]. MGMT methylation and/or low expression have been correlated with response to alkylating agents such as temozolomide and dacarbazine. MGMT expression levels have been incorporated into numerous molecular profiling assays, which may help clinicians identify patients with advanced HCC who are most like to benefit from ABT-888 and TMZ therapy.

HCC is a highly vascularized tumor that relies on a perfuse supply of blood for growth and metastasis. Human fibroblasts exposed to hypoxic conditions demonstrated compromised repair of double-strand DNA breaks, resulting in increased chromosomal instability [32]. It may be worthwhile to combine ABT-888 and TMZ with locoregional therapies such as TACE or trans-arterial radioembolization (TARE), which physically obstruct the arterial blood supply of tumors and potentiate tumor hypoxia.

In conclusion, the combination of TMZ and ABT-888 is relatively well tolerated in patients with advanced HCC. However, the regimen failed to demonstrate clinical activity in unselected patients. Predictive biomarkers that allow selection of patients who will respond to combination ABT-888 and TMZ should be further investigated in a variety of cancer types, including HCC.

## Electronic supplementary material

Supplementary material 1 (PPTX 82 kb)

Supplementary material 2 (PPTX 83 kb)

Supplementary material 3 (PPTX 83 kb)

Supplementary material 4 (PPTX 83 kb)

Supplementary material 5 (PPTX 85 kb)

Supplementary material 6 (PPTX 85 kb)

Supplementary material 7 (PPTX 91 kb)

Supplementary material 8 (PPTX 111 kb)
