# Supplementary material for: Phase II study of temozolomide and veliparib combination therapy for sorafenib-refractory advanced hepatocellular carcinoma
Source: Cancer Chemother Pharmacol. 2015 Oct 8;76(5):1073–9. doi: 10.1007/s00280-015-2852-2 (PMC4612326; doi:10.1007/s00280-015-2852-2)
Supplement: Supplementary file 6 — Supplementary material 6 (PPTX 85 kb) [file 280_2015_2852_MOESM6_ESM.pptx]

## Slide 1
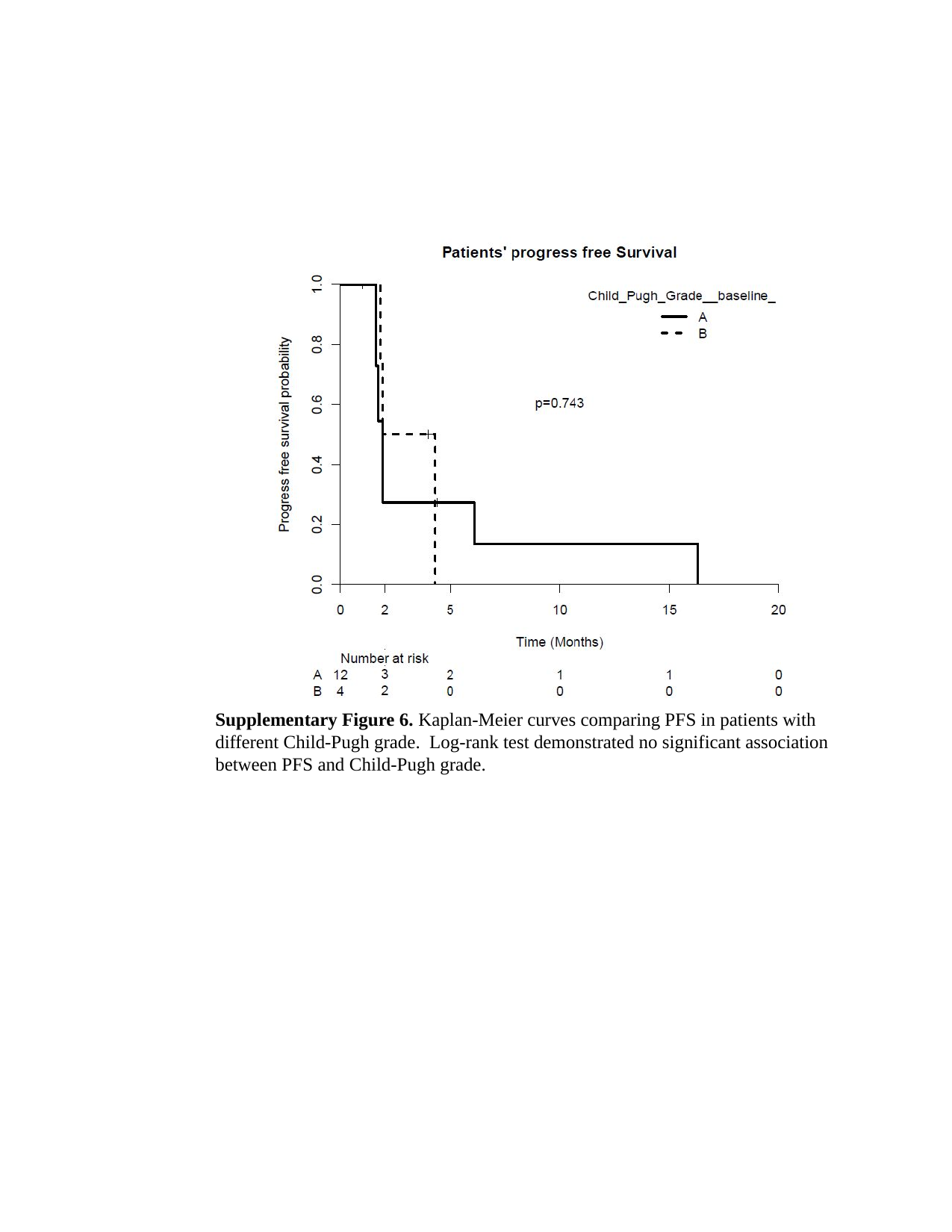

Supplementary Figure 6. Kaplan-Meier curves comparing PFS in patients with different Child-Pugh grade. Log-rank test demonstrated no significant association between PFS and Child-Pugh grade.
